# Approaches to ensuring and improving quality in the context of health system strengthening: a cross-site analysis of the five African Health Initiative Partnership programs

**DOI:** 10.1186/1472-6963-13-S2-S8

**Published:** 2013-05-31

**Authors:** Lisa R Hirschhorn, Colin Baynes, Kenneth Sherr, Namwinga Chintu, John Koku Awoonor-Williams, Karen Finnegan, James F Philips, Manzi Anatole, Ayaga A Bawah, Paulin Basinga

**Affiliations:** 1 Partners In Health. Boston, MA, USA; 2 Harvard Medical School, Boston, MA, USA; 3 JSI Research and Training, Boston, MA, USA; 4 Mailman School of Public Health, Columbia University, New York, NY, USA; 5 Department of Global Health, University of Washington, Seattle, WA, USA; 6 Centre for Infectious Disease Research in Zambia, Lusaka, Zambia; 7 Upper East Regional Health Directorate, Bolgatanga, Ghana; 8 Inshuti mu Buzima; 9 Brigham and Women’s Hospital, Boston, MA, USA; 10 School of Public Health, National University of Rwanda, Kigali, Rwanda; 11 The Bill & Melinda Gates Foundation, Seattle, WA, USA

## Abstract

**Background:**

Integrated into the work in health systems strengthening (HSS) is a growing focus on the importance of ensuring quality of the services delivered and systems which support them. Understanding how to define and measure quality in the different key World Health Organization building blocks is critical to providing the information needed to address gaps and identify models for replication.

**Description of approaches:**

We describe the approaches to defining and improving quality across the five country programs funded through the Doris Duke Charitable Foundation African Health Initiative. While each program has independently developed and implemented country-specific approaches to strengthening health systems, they all included quality of services and systems as a core principle. We describe the differences and similarities across the programs in defining and improving quality as an embedded process essential for HSS to achieve the goal of improved population health. The programs measured quality across most or all of the six WHO building blocks, with specific areas of overlap in improving quality falling into four main categories: 1) defining and measuring quality; 2) ensuring data quality, and building capacity for data use for decision making and response to quality measurements; 3) strengthened supportive supervision and/or mentoring; and 4) operational research to understand the factors associated with observed variation in quality.

**Conclusions:**

Learning the value and challenges of these approaches to measuring and improving quality across the key components of HSS as the projects continue their work will help inform similar efforts both now and in the future to ensure quality across the critical components of a health system and the impact on population health.

## Background

**Figure 1:**
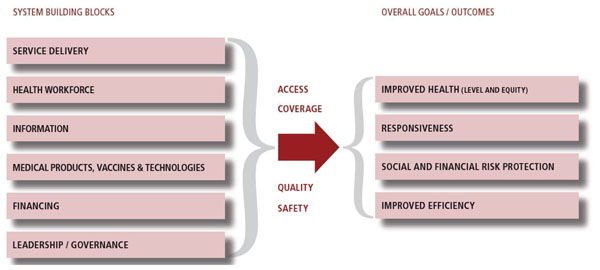
**Adapted from the WHO Framework** (Note: quality as central to overall goals) [1]

Reduction in population morbidity and mortality can only be realized through increasing not just access to needed services, but also the quality of services provided. Quality health services are defined as “effective, safe, centered on the patients needs and given in a timely fashion [1].” As work continues to focus on strengthening health systems, understanding how to best measure and improve quality is critical. The World Health Organization (WHO) focuses on quality as a central component of their framework on health system strengthening (HSS) and a key driver to ensuring that work to strengthen systems translates to improvement in health (Figure 1). Ensuring that quality is incorporated as a critical component of each of the six health system building blocks (service delivery; health workforce; health information system; medical products, vaccines and technologies; health financing; and leadership and governance), either implicitly or explicitly, will be essential to achieving potential health impact.

Other organizations and programs have highlighted the role and relevance of quality in both HSS and in achieving the overall goal of improved health. The United States Agency for International Development (USAID)-funded Health Systems Strengthening II project includes quality of care as one of its key components, with defined elements of quality going beyond care to include other key building blocks of the health system. These include improved planning, presence of supervision and management, supply chain, and strengthened referral systems [2]. Work to strengthen knowledge management, including data quality for evidence-based decisions and identification of gaps has also been identified as a core quality area in the context of HSS [3].

To ensure that these goals of quality are attained, there has been growing effort around quality improvement (QI) primarily focused on improving three of the six building blocks: service delivery; the health workforce; and the supply chain of medical products, vaccines, and technologies [4,5]. Leatherman and colleagues proposed mechanisms through which QI principles and methodologies could both strengthen health systems and further drive improvement in health outcomes. (Table 1) [6].

**Table 1:** Role of quality improvement (QI) in health systems strengthening using the WHO six building blocks framework (from Leatherman et al [6].)

Service delivery:	QI closes the gap between actual and achievable practice.
Health workforce:	QI enhances individual performance, satisfaction and retention.

Information:	QI enhances the development and adoption of information systems.

Medical products, vaccines and technology:	QI improves the appropriate, evidence-based use of limited resources.

Financing:	QI helps optimize the use of limited resources and helps reduce the costs of financial transactions.

Leadership and governance	QI strengthens measurement capacity, stewardship, accountability and transparency.

Despite the call for increased HSS efforts and the growing literature on quality improvement, there has been less written about feasible and effective approaches to measuring and improving quality in the context of HSS, particularly in areas outside of clinical care, data management, and supply chain. To help with measurement, a critical step for quality improvement, WHO provides a framework for monitoring and evaluating HSS efforts, which includes quality indicators for each building block [7]. However, there is not much known about the optimal set of quality measures to ensure that the strengthened systems and their associated services will be effective in improving population health.

In 2009, the Doris Duke Charitable Foundation’s African Heath Initiative (AHI) funded Population Health Implementation and Training (PHIT) Partnerships in five countries (Ghana, Mozambique, Rwanda, Tanzania, and Zambia) to improve population health through health system strengthening. While each Partnership developed a different approach to HSS, they were all designed to improve population health through increased quality and utilization of essential primary health care services within the public sector. The PHIT funding supported implementation of the different interventions and rigorous monitoring and evaluation, including measuring indicators of quality. A core set of AHI measures were chosen for tracking across the five sites, complemented by a unique set of measures developed by each Partnership, reflecting the main areas of focus and anticipated outcomes of their interventions.

We describe both the shared and program-specific approaches to measuring and improving quality among PHIT grantees. Understanding how the Partnerships are integrating the measurement and improvement of quality into their HSS efforts will be of value for other programs focused on improving health systems and population health at the district, provincial, or national levels.

## Description of approaches

The Partnership implementation plans were all focused on the WHO building blocks and are described in more detail in other papers in this supplement [8-12]. Therefore, we use the WHO HSS building blocks framework to explore the Partnerships’ approaches to ensuring quality as an essential outcome required to achieve improvements in population health. The framework was developed based on a review of selected literature on quality in the context of health system strengthening and authors’ experience in this field. Information from each Partnership program was obtained through a number of methods: PHIT project document review, semi-structured interviews with Partnership leads, and feedback during a consultative retreat with the Partnership principal investigators.

## Quality as a core principle in the PHIT projects

Each country Partnership is implementing a set of interventions largely based in the public sector and designed to strengthen health services and improve population health. The study designs are described in detail in related articles.[8-12]. The level of the health system targeted (community, facility, district, province) and planned activities vary across the Partnership. All Partnerships have identified and prioritized quality in their program implementation as both a primary outcome and as a critical step towards achieving health system improvements and public health impact. Work to measure and improve quality is integrated into the work to strengthen health systems and crosses most or all of the WHO building blocks.

The Partnership impact evaluation designs target the measurement of improvements in population health, including under-5 mortality. The designs also measure changes in quality related to the improvement initiatives implemented [8-12]. Measuring the relative importance of improving quality in achieving these population health gains is also integrated into the impact evaluation in most of the Partnerships. For example, in the Rwanda project, the impact evaluation framework is based on a theory of change where quality is a necessary component of each step, from input to processes to outcomes, to achieve improved population health and strengthened health systems. Areas where impacts on improving quality are measured include quality of care provided as well as quality of the health systems targeted for strengthening.

The Tanzania project framework is based on ‘realist evaluation’ tenets [13] with quality measured as an outcome and as a factor which influences supply and demand of services and informs further program implementation. In this framework, service quality is both an independent and descriptive outcome as well as a dynamic process which arises from the confluence of circumstance and program strategy. The Ghana framework predicts that addressing a number of gaps ranging from logistics, to access to data use is necessary to achieve the targeted population improvements [12]. However the Ghana Partnership includes quality as a critical factor needed within each intervention component for impact, whether it is the care provided by community volunteers, the quality of health information systems, or governance and decision making. In Zambia, improvement of quality is central to the anticipated impact. Their framework predicts that the intervention, based on facility-based mentoring and continuous QI, will improve clinical service quality, resulting in increased community value and utilization of care [8]. The diversity in the Partnerships is evident in the range of approaches used to ensure quality. The main areas of focus are summarized in Table 2. However, despite the heterogeneity of the Partnership implementation plans, there are areas of commonality for generating, measuring, and improving quality, which are discussed below.

**Table 2:** Core approaches to ensuring quality in the Partnerships

**Ghana**[12]: Strengthening information systems and tools and building management and leadership capacity to increase data-driven decision making and resource allocation.
**Rwanda**[10]: Improving population health through improving quality of clinical care, data quality and utilization, and underlying infrastructure and resources needed to deliver health services.

**Mozambique**[9,16]: Strengthening data-driven decision making and resource allocation by improving availability and quality of data as well as data utilization through capacity building and development of decision-support tools.

**Tanzania**[11]: Improving the quality of community-delivered services through contextually appropriate and people-centered care and improved information on community health status and needs.

**Zambia**[8]: Improving the quality of patient-provider interaction through targeted performance indicators that guide clinical mentorship teams and community health workers.

## Approaches to improving quality

Specific areas of overlap in improving quality fall into four main categories: 1) Defining and measuring quality; 2) Ensuring data quality and building capacity for data utilization for decision making and response to quality measurements including use of QI methodology; 3)Strengthening supportive supervision and/or mentoring; and 4) Conducting operational research to understand the factors associated with observed variation in quality (Tables 3 and 4). This approach reflects the Leatherman framework, which describes the role of QI in HSS, with Partnerships focusing on the building blocks to improve quality as defined within each component (Table 3). Many of the efforts overlap with more than one building block and so expected outcomes capture numerous impacts of QI. For example, the focus on improving data quality and use is expected to result in more effective, evidence-based use of limited resources (medical products, vaccines, and technology building block) and measurement capacity, stewardship, accountability, and transparency (leadership and governance building block) (Table 4) [6].

**Table 3:** Selected definitions of quality in WHO HSS building blocks and Individual PHIT Partnerships

	Service delivery	Health Workforce	Information	Medical Products, Vaccines, & Technologies	Financing	Leadership and governance
WHO [1]	Coverage, comprehensive accessibility continuity, person-centeredness coordination, accountability and efficiency General and specific service readiness score ^1^	Health worker distribution*	Performance of specific surveys and other health measurements Facility reporting Data quality (through Data quality audits: DQA)	Availability of tracer drugs*	National expenditure of health Out of pocket expenditures Insurance coverage	Presence of relevant strategies and guidelines

Ghana [12]	Quality of care delivered by CHWs Patient satisfaction Community satisfaction (care (availability, perception of quality)	Full complement of staff per facility Supervision of CHWs by DHMTs	Effective use of data to drive appropriate allocation and care delivery Data quality (concordance)	Availability of tracer drugs and other commodities	Allocation of project funds reflective of identified needs	Leadership capacity Data-driven allocation of funds

Mozambique [16]	Timeliness of primary health care service provision Patient satisfaction Population coverage Service integration	Efficiency in the allocation of trained health workers Availability of trained health workers. Frequency of supervision visits (facility and district).	Data quality (through DQA)	Availability of tracer drugs and other commodities	Equity of funding distribution across districts Public sector capacity for management of project funds Financial management capacity	Availability of trained district and facility management personnel Frequency of management meetings (district and facility)

Rwanda [10]	Quality of care delivered Service volume Population coverage (equity; effective coverage)	Facility staffing Staff training HCW retention and satisfaction Receipt of supervision	Data quality (focus on health facility and CHW registries) Internet/network downtime Utilization of facility data for management decisions	Appropriate equipment levels Availability of tracer drugs and other commodities Strength of district supply chain management Availability of selected lab capacity	Insurance coverage Costing of services delivered Public sector management of project funds	Utilization of data to drive improvement

Tanzania^2^[^11^]	Availability of selected services, Outreach performed for care Quality of care	Staff training Facility staff levels Receipt of supervision Performance of QA activities	Required routine data reports submitted	Availability of tracer drugs and other commodities Availability of selected lab capacity		Meetings at Health Facility to discuss management and governance

Zambia [8]	Quality of care delivered Service readiness Guideline availability at site Community reported utilization of selected health services	Density, motivation and training of health workers	Data quality and record keeping	Availability of selected tracer drugs and other commodities	Financial planning capacity and activities Public sector management of project funds Geographic equity of funding allocation	Facility governance (self-rated) Community participation in health service delivery and perceived appropriate governance Funding allocation and activities reflecting identified gaps

WHO: World Health Organization; CHW: community health workers; DHMT: District Health Management team; QA: Quality Assurance

*chosen as core measures across all PHIT projects

^1^ Includes drugs and commodities infrastructure, (basic amenities), basic equipment, laboratory, infection control and specialized services

^2^ Also used principal component analysis to convert data from MACRO Service Provision assessment tool (SPA) into composite indices of health system strength (e.g. readiness to provide curative care, readiness to provide preventive care, readiness to provide advanced clinical care)

**Table 4:** Specific interventions for improving quality in selected areas in the PHIT programs

Country	Area of focus as described by Partnership (main WHO building block)*	Interventions
Ghana [12]	Information management (I, MVT)	Implementation of a “simplified register” that condenses the volume of registers that workers manage each month from 28 to five, greatly reducing the burden of data capture and simplifying the process of information reporting. Development and implementation of a District Health Planning and Reporting Toolkit Utilization of simplified logistic monitoring tools to strengthen capacity to monitor status of supply readiness at service delivery points.

	Logistics gap (MVT)	Employment of simple logistics monitoring tools developed in Nkwanta district for the PHIT-supported initiative to allow district teams monitor supply readiness at all service delivery points.

	Leadership capacity (LG)	Leadership and management training to build capacity of district and sub-district managers to better manage and supervise frontline healthcare personnel; utilize data for decision making, and strengthen planning and decision making for resource allocation.

	Evidence-based resource allocation and other decision making (LG)	Management training to ensure utilization of the District Health Planning and Reporting Toolkit and other data for decisions and resource allocation.

Mozambique [16]	Improved systems and quality of care (I, LG)	Improved data-driven decision making capacity through: development of appropriate tools to facilitate decision-making for provincial and district managers (quarterly report card/data dashboard that provides longitudinal comparisons of key PHC indicators across all facilities within a district and across all districts within the province); strengthening of data-driven decision making through capacity-building in management and leadership including linking data with annual planning, and combination of in-service trainings and post-training coaching focusing on problem identification, solution generation, implementation, and assessment; applied research to understand and/or test innovations to overcome bottlenecks.

	Human resource allocation (HW)	Development of a simple optimization model to simulate and improve human resource allocation.

	Data systems, data quality and feedback loop (I)	Regular assessment including DQAs of availability, consistency, accuracy and validity of data for key primary health care system Monthly review by district staff for data quality with rapid feedback to address gaps.

Rwanda [10]	Quality of clinical care and supervision (HW)	Training of Heath center nurses followed by ongoing mentoring and enhanced supportive supervision (MESH) from nurse mentors. Mentors also help identify and address system barriers to care through coaching in quality improvement.

	Data quality and utilization (I, LG)	Partnership with the MOH to perform DQAs with support ongoing to address and improve data quality. Support of district, facility and community heath staff to utilize data through training, reports development and other decision aids (e.g. dashboards).

	Infrastructure and supplies (SD, MVT)	Provision of infrastructure support based on measured gaps between existing resources and MOH guidelines at the health center with follow-up monitoring. Strengthening of district pharmacy and ongoing monitoring and feedback on stock-outs and equipment gaps.

Tanzania [11]	Equity of access to and receipt of needed services (SD)	Training and deploying of Community Health Agents to deliver community-based reproductive, maternal, newborn and child health promotion services as an integrated package of community-based primary care.

	Supervision and governance (LG)	Strengthening supervisory systems and community governance mechanisms.

	Referral systems (SD)	Development and implementation of a referral system through training and infrastructural improvement to improve accessibility.

	Information systems and utilization (I, MVT)	Launch of information and monitoring operations and implementation of logistics support systems.

Zambia[8]	Quality of clinical care (SD, HW)	Training and intensive clinic mentoring by district clinical quality teams Implementation of practical tools that establish clear clinical care standards supported by initial training and mentoring.

	Supervision (SD, HW)	Supportive reinforcement of the standards through ongoing supervision and mentoring by the district clinical quality team.

	Resources (MVT)	Ensuring sufficient resources including medicines and equipment needed to deliver care according to standards.

	Data utilization (I)	Implementation of a performance feedback loop based on information from clinical management tools. Clinic performance measurement reports are produced and in use by QI teams to support clinician and health center mentoring and supervision and identify health system gaps contributing to lower performance.

	Community participation in health (SD)	Training and deployment of community health workers with skills to promote available services at the facilities and adherence to recommended care and to recognize danger signs and make timely referrals Measurement of community perceptions of appropriate governance.

	District capacity for quality measurement and supervision (SD,MVT,LG)	Supporting district-based staff, including the QI teams, a community coordinator, and a pharmacy technician.

*SD: Service delivery; HW: Health workforce; I: information; MVT Medical Products, Vaccines, & Technologies; F: Financing; LG: Leadership and Governance

## Defining and measuring quality in the context of health systems strengthening interventions

At the start of the AHI initiative, agreement was reached to collect a selected number of core measures of quality across all Partnerships (described in more detail in the paper by Bryce et al. in this supplement) [14]. Two of these – health workers per capita and continuous stocks of essential commodities – reflect WHO guidance on monitoring and evaluation of HSS [7]. Other core measures that capture aspects of quality include specific coverage measures (e.g., intermittent preventive treatment for malaria in pregnancy, skilled attendant at birth, and appropriate management of selected childhood illness). Despite significant discussion and brainstorming, there remain a number of areas where consensus on specific indicators of quality within one or more of the HSS blocks could not be reached. As a consequence, the teams agree to report on their Partnership-specific activities to improve quality of care and measure quality annually.

In each project, quality is defined, measured, and targeted for improvement across many or all of the six WHO HSS building blocks, going well beyond the traditional focus on service quality. The measurement of the impact of these improvement efforts on quality is integrated into each Partnership impact evaluation plan. Data sources in use by the PHIT Partnerships for measuring quality fall into three main categories: 1) use of routinely available data (e.g., facility reports and health management information system (HMIS); 2) data collection tools developed within the intervention and integrated into routine project monitoring (e.g., facility surveys and mentor reports); 3) and measurement solely for interim and summative impact evaluations designed for baseline measurement, mid-course correction, and end-of-project assessment.

Despite the heterogeneity of interventions and independence of evaluation designs, there is significant overlap in approaches targeting the definition of quality (Table 3). While some of these measures overlap with WHO recommendations on measuring components of quality, others reflect the specific pathways through which each Partnership is working to strengthen the health system and improve population health.

### Service delivery

The service delivery building block has the greatest range of measures of quality across the Partnerships. Many measures reflect the WHO recommendations around access, coverage, and patient-centeredness. Components include availability of services, reported utilization and coverage (access), quality of care per national protocols, timeliness, and patient and community satisfaction (patient-centered). This includes services delivered at facilities and by community-based health workers.

### Health workforce

All the Partnerships measure and work to improve health care worker distribution and density and supervision activities as fundamental components of a human resource strategy. These are core components for Rwanda and Zambia, which are implementing a mentoring model for health facility staff. A few projects also target additional factors, including staffing levels at facilities, management of staff, retention levels, satisfaction, motivation, and the quality of care provided. A number of the Partnerships (Tanzania, Rwanda, and Ghana) also include a focus on strengthening the community health workers workforce through ensuring adequate staffing levels, training, and supervision of this cadre. To improve management and leadership skills of district health managers and their teams as key members of the health workforce, the Ghana team, with support from UNICEF, developed tools that were used to train all district health managers in the operational areas of the project. Mozambique measures the efficiency of allocating trained staff as an additional component of human resource system quality.

### Information

Aligned with WHO priorities, ensuring data quality is the most common activity across projects, although specifics vary [15]. The PHIT Partnerships all focus on both the processes of measuring data quality and the level of data quality attained. Data utilization for decision making is a core component of quality within this building block, as well as attributes of the data utilized, such as timeliness and accuracy of required reporting.

### Medical products, vaccines, and technologies

Based on WHO recommendation, a core indicator across the Partnerships is the availability of a set of tracer drugs and other commodities to assess health service readiness at the facility or community health worker (CHW) levels. However, the specific list of drugs and other commodities are tailored to reflect country guidelines. Additional measurement areas include equipment levels and overall strength of the district supply chain management system and availability of lab testing capacity.

### Financing

Partnerships focus on assessing the quality of financial management, including the use of the PHIT project funds to support the planned activities. Three of the projects explicitly identify equitable or data-driven allocation of resources as a measure of quality of financial systems (overlapping with measures of quality of governance) (Table 4). In Rwanda, insurance coverage (a WHO identified area) and cost is measured as an additional area of financial quality.

### Leadership and governance

While WHO focuses on the presence of relevant strategies and guidelines largely at the national level, the Partnerships are a practical operationalization of the governance and leadership building block at the provincial, district, or lower levels, with an emphasis on how systems are governed and managed locally. Ghana and Mozambique explicitly measure governance, focusing on collecting documentation of management and evidence-based allocation of resources including use in Ghana of a tool specifically designed to enable managers to make budget decisions based on need rather than previous allocations. Community participation and their perceived levels of good governance are measured in Zambia as an indicator of quality governance.

## Ensuring data quality and building capacity for data utilization

A major theme across the Partnerships is the increase of service quality through strengthening data utilization for evidence-based decision making, as well as the identification and addressing of gaps and allocation of resources more efficiently (Table 4). Work includes efforts such as ensuring strong feedback loops to increase capacity of end-users for data analysis, interpretation, and communication. However, every project also recognizes the need to ensure the robustness of HMIS data as a core component in this approach to generating and ensuring quality.

### Ensuring and improving data quality

Measuring and improving data quality is a core theme across the projects. Activities include routine measurement using data quality audits (DQAs), data checks integrated into electronic health information systems, and routine reviews by supervisors. Most data quality-improvement efforts focus on concordance with primary sources as well as completeness and correctness. Efforts to improve data quality are also integral to Partnership activities and include both direct activities (e.g., training and supervision of data management staff, feedback of data quality measurement results, and supervision), as well as indirect activities where data quality is improved by feedback of performance results and reliance on data for resource allocation and other management decisions.

For example, in Ghana, tools have been developed to capture information on Community-based Health Planning and Services (CHPS) scale-up in the Upper East Region. The electronic database capture system developed has rigorous data checks built into the database to ensure data consistency and integrity. Independent verification procedures are also in place to ensure that data captured are of the highest quality.

In Mozambique, improving data quality is an explicit Partnership goal and is considered a necessary process for improving governance and financing through data-driven resource allocation.

Work on improving data quality complements the efforts to ensure effective data utilization for HSS. Activities to measure and improve data quality focus on improving data collection, DQAs, analysis of HMIS gaps at facility and district levels, and feedback of these results through easily interpreted flyers to improve quality. Support includes building skills needed to improve HMIS functioning (electronic and paper-based systems), training for performing routine data quality assessments, and instruction and coaching on how to understand and use the results. Rwanda actively partners with the district health team to perform DQAs and strengthen data quality through increasing data feedback and utilization.

In Tanzania, community health agents (CHAs) complete monthly reports, which are reviewed before feedback is given on potential data quality gaps. In-service trainings are then provided on HIS data collection and reporting focuses on the identified gaps. Lessons learned from the first group of CHAs were incorporated into the training of the second group with the goal of improving and sustaining health information quality.

The Zambia Partnership assesses data on selected services from an electronic data capture system for completeness as a routine component of data management, ensuring that data used for feedback accurately reflect the services delivered. Similar work is underway in Rwanda through the improvement of the electronic medical record (EMR) data using integrated electronic data checks and routine DQA for concordance with paper charts. Work is also underway to improve routine paper-based MOH reports compiled by trained data coordinators who partner with district supervisors. This is conducted to measure concordance of reported aggregate data with health center registries and provide support to health center and district managers to strengthen feedback and data improvement activities.

### Strengthening data utilization

In Ghana, training to improve supervision capacity also includes a focus on data interpretation. Data are fed back to regions, districts, and sub-districts, with team leaders responsible for monitoring and evaluation being supported in data interpretation, systems analysis, and development of an action plan based on the information disseminated. This work is supported through the development and use of simplified health information registers (“simplified register”) and information management tools (e.g., logistics systems monitoring and the District Health Planning and Reporting Toolkit, DiHPART). For example, while future budgets had been based on previous budgets, DiHPART enables district managers to allocate budget priorities according to measured need.

Rwanda and Mozambique share a similar approach with trainings underway to develop analytic, interpretation, and communication skills for program managers, health center directors, and data managers. These are reinforced by regular meetings where results are presented and discussed. In Rwanda, PHIT-supported data coordinators also accompany the MOH data officers at sites to provide training and mentoring in the communication and use of DQA results. The Rwanda team also developed tools including dashboards and indices which facilitate data use by summarizing multiple data sources. These data are being used to allocate resources for health center infrastructure strengthening.

The Mozambique Partnership focuses on building similar skills to estimate coverage and analyze programmatic gaps. It then links these results with program and district budgets, and activity planning. In Tanzania, the program intervention reflects the knowledge that while there was experience in assessing and improving quality of services, skills were needed in analyzing data to understand and address the causes of service quality gaps. Work is underway to strengthen capacity to triangulate data collection and analysis aimed at assessing factors and processes which influence quality of community-based primary health care. There is also ongoing effort to better understand how variation in performance affects the health system.

## Strengthening supportive supervision and mentoring

As Leatherman points out, QI can serve to decrease the “gap between actual and achievable practice” for service delivery and to enhance “individual performance, satisfaction and retention” for health workers [6]. Every project includes a component of supportive supervision or mentoring as a means to directly bridge that gap while building capacity among staff at the health facility, district, or province level to better respond to identified gaps.

In Rwanda, the project includes a health center nurse mentoring and supervision program, which provides direct feedback through observation of care in four main areas: outpatient under-5 care; outpatient adult care; HIV and TB care; and maternal health care, focusing on antenatal, labor, and delivery services [10]. The nurse-mentor also provides coaching to the care teams at the health centers to identify and address gaps in quality using systems-based quality QI methodology. Program managers are also given training and mentoring in QI methodology to drive improvement in other areas including inpatient and specialty care and support systems (e.g., pharmacy, information systems etc.). In Zambia, onsite clinical mentoring of health care workers is among the core essentials of the intervention. A team of qualified clinicians uses automatically generated performance indictors to support data utilization. This, in turn, improves the quality of provider-delivered care [8].

The Mozambique Partnership focuses on increasing supervision quality and frequency to better integrate and improve service management at the facility, district and provincial levels. Steps to improve supervision quality include the introduction of a data collection guide for supervision visits to ensure that data quality weaknesses and primary health care utilization gaps are identified. Together with additional data summary tools, these results are discussed with district and facility managers to identify priority problems and define an action plan which is also used for follow-up in subsequent supervision visits.

The Tanzania Partnership links each CHA with both a clinical supervisor based at the health center and a community-based supervisor. Supervisors meet with the CHAs regularly to ensure that CHA responsibilities are met and supported by a strong feedback loop of both activity and interim evaluation data on activities. The process of supervision is improved through feedback of results from interviews with CHA and checklists to measure the quality and frequency of this activity. The results are used to guide the project on planning, management, supervisory decisions, and steps needed to address gaps in the quality of community-based primary healthcare and in the supervision. In the Ghana Partnership, supervisor training is focused on improving supervision capacity. Relevant data are also fed back to the region and then to the districts or sub-districts to further strengthen supervision, ensuring that the district team lead is an important part of the monitoring and supervision processes.

## Operational research on quality

The Partnerships incorporated operational research into the program design to measure and provide insights on the level of quality in the building blocks, the factors associated with variations of quality, and the impact of this variability in achieving population health goals. In Ghana, pilot research is used to guide implementation activities. For instance, a referral scheme to improve maternal and child health is being implemented in one sub-district in one of the intervention districts. Lessons learned from this pilot are guiding scale up efforts in different districts. In Mozambique and Rwanda, this work is combined with in-country research capacity building efforts. In Mozambique, applied research methodologies are used to understand and test innovations to overcome system bottlenecks. The Mozambique Partnership provides financial and technical support to the Ministry of Health’s Beira Operations Research Center (CIOB), as well as masters-level public health training, to build sustainable applied research capacity [9,10]. In Rwanda, scholarships are provided to field- or MOH headquarter-based implementers to pursue advanced degrees while completing research projects. Research project address factors associated with differences in the quality and the effectiveness of approaches to improve quality of care, services, human resources, and other components of HSS.

## Conclusions

While the PHIT initiative is focused on HSS to improve population health, all projects focus on quality across the WHO building blocks as a key outcome and necessary step to achieving the longer term impact. Each of the Partnerships uses different approaches and theoretical frameworks to strengthen health systems and ensure quality; however, they all include work on measuring quality, improving data quality and building capacity of end users so that results identify and address gaps in quality.

Many of the efforts to measure quality are integrated into routine work and monitoring and evaluation rather than existing as separate data collection efforts for performance measurement or evaluation. As a result, all the Partnerships recognize the need and challenge of ensuring that these data are of adequate quality – a priority reflected in the work of building systems and capacity for data quality. In addition, using these data for evidence-based decision making is used as a strategy to strengthen other building blocks, most prominently in leadership and governance. The approach to improve identified gaps in quality varied across Partnership projects, but all recognize the need for strong, supportive supervision, and mentoring. This includes direct mentoring of care providers, mentoring and training of managers to respond to system gaps – including resource allocation – and ongoing adaptations of the interventions to increase quality and in turn improve expected impact. Comparing the approaches with the Leatherman description of the role of QI in HSS, the cross-building block- QI approach to strengthen health systems captures almost all of the potential benefits regardless of the program analyzed [6]. The Partnerships measures capture these areas of improvement as well as additional areas within the building blocks identified by the projects as critical to achieving a quality health system able to improve population health. A challenge for the PHIT Partnerships is to identify areas of common intervention and measurement where lessons learned can be shared and approaches compared within the constraints of the different intervention models and settings. Work to develop focused cross-Partnership evaluations in areas such as mentoring and strengthening data utilization are under discussion.

In conclusion, measuring and ensuring quality beyond the health care delivery building block is identified by all PHIT Partnerships as core to success, with an integrated approach to ensuring a robust feedback loop for improvement. Projects are prioritizing different areas and levels for this work but core themes include a focus on data quality and building capacity to use these data to identify gaps and improve quality more effectively and efficiently. The impact evaluation in each Partnership is designed to capture improvement in quality across multiple HSS building blocks in the context of the implementation plans. Results from the operational research and impact evaluations planned by each Partnership will help increase the understanding of the effectiveness of HSS efforts on improving quality in care delivery and such other critical areas as leadership and health information systems. The Partnership projects will also contribute to the documentation of the relative importance of these improvements on measured changes in population health. The challenge for each country project will be to identify the optimal approaches to improving and maintaining quality within each of the building blocks in order to achieve an effective, strengthened and sustainable health system.

## List of abbreviations used

AHI: African Health Initiative; CHAs: Community health agents; CHPS: Community-based Health Planning and Services; CHW: Community health worker; CIOB: Ministry of Health’s Beira Operations Research Center; DHMT: District Health Management Team; DiHPART: District Health Planning and Reporting Toolkit; DQA: Data quality audits; EMR: Electronic medical record; HMIS: Health management information system; HSS: Health systems strengthening; PHIT: Population Health Implementation and Training; QA: Quality assurance; QI: Quality improvement; UER: Upper East Region; WHO: World Health Organization.

## Competing interests

The authors declare that they have no competing interests.

## Declarations

This article has been published as part of *BMC Health Services Research* Volume 13 Supplement 2, 2013: Improving primary health care to achieve population impact: the African Health Initiative. The full contents of the supplement are available online at http://www.biomedcentral.com/bmchealthservres/supplements/13/S2. Publication of this supplement was supported by the African Health Initiative of the Doris Duke Charitable Foundation.
